# Music improves social communication and auditory–motor connectivity in children with autism

**DOI:** 10.1038/s41398-018-0287-3

**Published:** 2018-10-23

**Authors:** Megha Sharda, Carola Tuerk, Rakhee Chowdhury, Kevin Jamey, Nicholas Foster, Melanie Custo-Blanch, Melissa Tan, Aparna Nadig, Krista Hyde

**Affiliations:** 1 International Laboratory for Brain, Music and Sound Research (BRAMS), Department of Psychology, University of Montreal, Pavilion Marie-Victorin, 90 Avenue Vincent D’Indy, Montreal, QC H2V 2S9 Canada; 2 Centre for Research on Brain, Language and Music (CRBLM), Faculty of Medicine, McGill University, Rabinovitch House, 3640 de la Montagne, Montreal, QC H3G 2A8 Canada; 3 Westmount Music Therapy, 4695 Maisonneuve Boulevard West, Westmount, QC H3Z 1S4 Canada; 4 School of Communication Sciences and Disorders, Faculty of Medicine, McGill University, 2001 Avenue McGill College, Montréal, QC H3A 1G1 Canada

## Abstract

Music has been identified as a strength in people with Autism Spectrum Disorder; however, there is currently no neuroscientific evidence supporting its benefits. Given its universal appeal, intrinsic reward value and ability to modify brain and behaviour, music may be a potential therapeutic aid in autism. Here we evaluated the neurobehavioural outcomes of a music intervention, compared to a non-music control intervention, on social communication and brain connectivity in school-age children (ISRCTN26821793). Fifty-one children aged 6–12 years with autism were randomized to receive 8–12 weeks of music (*n* = 26) or non-music intervention (*n* = 25). The music intervention involved use of improvisational approaches through song and rhythm to target social communication. The non-music control was a structurally matched behavioural intervention implemented in a non-musical context. Groups were assessed before and after intervention on social communication and resting-state functional connectivity of fronto-temporal brain networks. Communication scores were higher in the music group post-intervention (difference score = 4.84, *P* = .01). Associated post-intervention resting-state brain functional connectivity was greater in music vs. non-music groups between auditory and subcortical regions (*z* = 3.94, *P* < .0001) and auditory and fronto-motor regions (*z* = 3.16, *P* < .0001). Post-intervention brain connectivity was lower between auditory and visual regions in the music compared to the non-music groups, known to be over-connected in autism (*z* = 4.01, *P* < .00001). Post-intervention brain connectivity in the music group was related to communication improvement (*z* = 3.57, *P* < .0001). This study provides the first evidence that 8–12 weeks of individual music intervention can indeed improve social communication and functional brain connectivity, lending support to further investigations of neurobiologically motivated models of music interventions in autism.

## Introduction

Autism Spectrum Disorder (ASD) is a neurodevelopmental condition characterized by social communication difficulties and restricted and repetitive behaviours among strengths in varied domains^1^. ASD is highly prevalent but there is considerable heterogeneity in its aetiology, clinical presentation and underlying brain connectivity^1,2^. Consequently, a variety of behavioural and psychosocial treatments are sought by families^3^. However, there is little consensus on which treatments are most effective^4^. Thus, a diagnosis of ASD is associated with substantial costs to the individual, the family and the community^5^.

ASD is a lifelong condition with a median age of diagnosis >4 years^6^, although most current intervention strategies target children <6 years to promote early behavioural change^3^. Individuals with ASD and their families face significant challenges during developmental transitions^7^. School-age children in particular often remain unengaged in social settings, reducing opportunities for socio-communicative development^8,9^. This has led to investigations of alternate and creative means of expression such as music that might improve social communication, increasing prospects for meaningful relationships. Furthermore, cross-culturally applicable music-based interventions hold potential for scalability at home, school and global community settings^10^.

Previous randomized controlled trials (RCTs) of music interventions for ASD have reported positive effects of music on emotional engagement, social interaction, communication and parent–child relationships^11,12^, suggesting that musical activities in a therapeutic context can promote measurable behavioural changes in children with ASD. Strengths in music processing have been noted since the first description of ASD^13^ and many studies have reported intact or enhanced musical skills such as absolute pitch, enhanced melodic memory and contour-processing in children with ASD^14–16^. Greater brain responses to song versus speech in fronto-temporal brain regions^17,18^ and intact emotional responsiveness to music have also been demonstrated^19^. Supporting anecdotal reports from parents and caregivers have described the profound effects music has had on children with ASD^20^.

The positive impact of music on social skills has been demonstrated beyond ASD^21,22^. Typically developing children are more likely to play with another following a shared musical experience^23^ and joint musical interactions can enhance emotional empathy, prosociality and bonding in children^24–26^. More recently, neuroimaging studies have shown that participating in musical activities engages a multimodal network of brain regions involved in hearing, movement, emotion, pleasure and memory^27–31^, thus allowing transfer of music-related therapeutic effects to non-musical domains^32^ through structural and functional brain changes^33,34^. However, a direct link between effects of music interventions and changes in the brain is yet to be demonstrated in autism^35,36^ and was our aim here.

Altered intrinsic brain connectivity is a hallmark of ASD. Both over-connectivity and under-connectivity have been reported, in particular, under-connectivity of fronto-temporal and cortico-subcortical networks and over-connectivity of sensory networks may be considered potential treatment targets^37–40^ given their associations with verbal and social communication skills in autism^18,41^. Resting-state functional magnetic resonance imaging (rsfMRI) allows measurement of intrinsic brain connectivity by computing temporal correlations of spontaneous blood-oxygen-level-dependent (BOLD) signals among spatially distributed brain regions and may be a promising target of music-induced neuroplasticity^40,42^. The use of resting-state functional connectivity (RSFC) as an outcome measure for intervention studies, particularly for clinical populations, has been recommended since it affords the advantage of being task-independent, has high test–retest reliability, limited practice effects and can provide reliable estimates of functional brain connectivity corresponding to underlying anatomy^43^.

Currently, evidence for effectiveness of music interventions is limited and there is no neuroscientific basis for its use in ASD. However, given the impact of music on social functioning and brain connectivity, alongside atypicalities in these areas in ASD, music-based activities may restore altered brain connectivity and social difficulties in ASD. Synthesizing findings from previous research, two possible mechanisms for such music-induced neuroplasticity and its impact on social functioning may be proposed:^32,36,44,45^ (1) top–down reward-based cortical modulation to reinforce learning of non-musical behaviours such as social interactions through the intrinsic reward value of music, (2) bottom–up sensorimotor integration through sound and auditory–motor entrainment of neural networks through synchronization leading to modulation of atypical sensory processing, which in turn may improve social communication^41,46^. Our goal was to investigate whether music-based interventions can indeed alter spontaneous rsfMRI signals, leading to improved functioning in ASD based on one of the above hypotheses.

The specific aim of this RCT was to investigate whether 8–12 weeks of a music-based intervention (compared to a non-music control intervention) can improve social communication, family quality of life (FQoL) and functional brain connectivity in school-age children with ASD. This would provide evidence for an effective, inexpensive, easy-to-administer and relatively non-specialized strength-based intervention that may be scaled in varied settings across cultures, addressing the need for globally applicable ASD intervention models^10^.

## Materials and methods

### Study design

We report an assessor-blinded, parallel-group RCT^47,48^ of a music intervention (MT) compared to a non-music control intervention (NM) for improving social communication and fronto-temporal brain connectivity in school-age children with ASD. The trial (isrctn.org: ISRCTN26821793) was conducted between April and December 2016 in Montreal, Canada with ethics approval from the Montreal Neurological Institute (MNI) at McGill University. Written informed consent was obtained from parents/guardians of participants.

### Participants

**Fig. 1:**
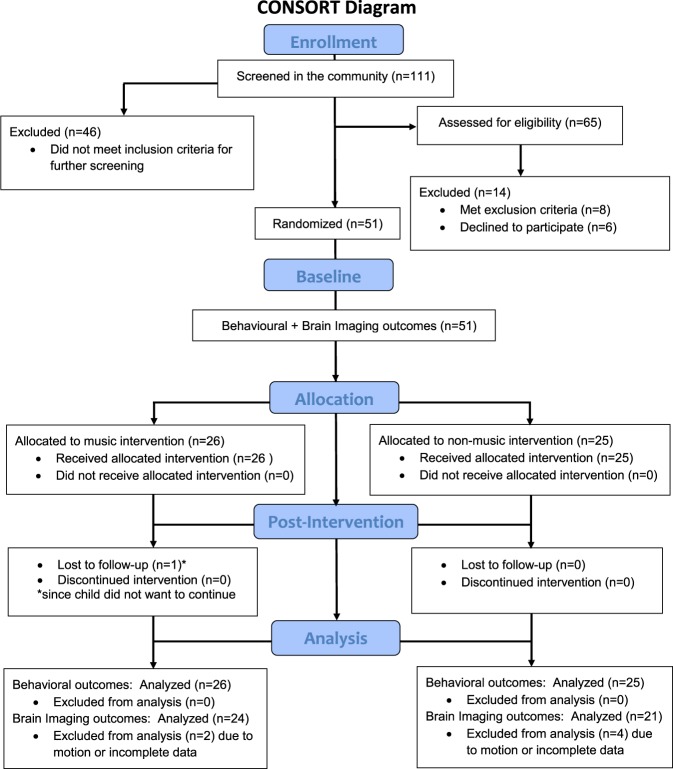
CONSORT study diagram. CONSORT study diagram study comparing neurobehavioural outcomes of a music intervention compared with a non-music intervention for children with Autism Spectrum Disorder

Children aged 6–12 years, meeting Diagnostic and Statistical Manual of Mental Disorders, Fourth edition criteria for ASD^49^, were screened from January to August 2016 (Fig. 1). Exclusion criteria were (1) individual music therapy within 6 months prior to study, (2) private musical lessons for a cumulative period of 1 year prior to study, (3) group music therapy in school; (4) <35 weeks of gestation, (5) hearing disorders or (6) a medical history of neurological disease. Power analysis using evidence-based effect size estimation^11^ suggested that for a large effect (*d* = 0.8) of music therapy on social communication, detectable with 80% power at *P* < .05, a sample of *n* = 50 (25 per arm) would be required.

### Baseline assessment

Assessment at baseline consisted of two sessions. In the first session, detailed demographics on socioeconomic status^50^ (SES), handedness^51^, music experience history and past and current intervention history of the child were obtained. Participant diagnosis was confirmed using a best-estimate diagnosis of ASD supported by an ADOS (Autism Diagnostic Observation Scale^52^), Autism Diagnostic Interview–Revised^53^ or Childhood Autism Rating Scale^54^ and detailed clinical assessment report. Additionally, parent-reported behavioural outcomes on Social Responsiveness Scale (SRS-II^55^), the Children’s Communication Checklist (CCC-2^56^), the maladaptive behaviour subscale of the Vineland Adaptive Behaviour Scales (VABS-MB^57^) as well as the Beach Family Quality of Life Scale (FQoL^58^) were obtained. Children’s cognitive ability was assessed using the Wechsler’s Abbreviated Intelligence Scale (WASI-II^59^). If the child had completed an intelligence quotient (IQ) test (WASI-I/II/WISC-IV/V) within 2 years of the study, available scores were used. Children’s language ability was assessed using the Sentence Repetition subtest of the Clinical Evaluation of Language Fundamentals (CELF-4^60,61^) and receptive vocabulary was measured using the Peabody Picture Vocabulary Test (PPVT-4^62^). Musical ability was assessed using the Montreal Battery for Evaluation of Musical Abilities^63^. Detailed baseline characteristics of participants are provided in Table 1.

**Table 1:** Baseline characteristics of participants

Measure	Music			Non-music			*P* value
	*n*	Mean	SD	*n*	Mean	SD	
*Participant characteristics*							
Age (in years)	26	10.30	1.91	25	10.20	1.87	.85
Sex (male:female)	26	21:5	0.40	25	22:3	0.33	.75
Social Communication Questionnaire	26	21.31	5.90	25	19.68	5.50	.31
ADOS total^a^	22	15.64	5.50	13	14.84	4.62	.65
Language impairment^b^	26	13/26	—	25	14/24	—	.53
Parent-reported sentence level speech	26	23/26	—	25	19/25	—	.29
Verbal IQ^c^	25	94.72	21.40	23	87.30	23.47	.26
Nonverbal IQ^c^	24	110.79	18.15	21	102.38	18.22	.13
Full-Scale IQ^c^	25	102.00	18.82	24	94.00	18.18	.14
MacArthur SES (Ladder)^d^	26	5.38	1.83	25	5.72	2.28	.57
Annual income (in $)	25	39760	30847	25	43300	30145	.68
Handedness (augmented laterality index)^e^	26	71.12	51.63	25	73.28	52.74	.88
Musical ability (MBEMA)^f^	23	0.72	0.14	22	0.69	0.14	.57
VABS gross motor skills^k^	26	13.5	2.2	25	13.24	2.08	.67
VABS fine motor skills^k^	26	15.92	3.09	24	15.21	2.6	.38
Number of therapy sessions completed	26	10.50	1.61	25	10.16	1.70	.47
*Outcome measures*							
SRS-II T-score^g^	26	70.15	9.62	25	72.24	11.43	.48
CCC-2 general composite^h^	25	76.84	14.44	23	77.65	13.35	.46
PPVT-4 standard score^i^	26	94.58	26.18	25	85.48	29.42	.25
Family Quality of Life^j^	26	102.38	13.62	25	104.08	13.79	.66
VABS maladaptive behaviours^k^	25	19.80	1.50	23	20.00	1.86	.69

*P* values are calculated using independent samples *t* tests for continuous variables and chi-square tests for categorical variables between groups

*SD* standard deviation

^a^ADOS: Autism Diagnostic Observation Scale Total score from ADOS or ADOS-2. Higher scores mean greater symptom severity

^b^Number of participants meeting criteria for language impairment based on scaled scores 1 SD or greater below the mean (=10) on the Sentence Repetition subtest of Clinical Evaluation of Language Fundamentals (CELF-4)^60,61^

^c^IQ was measured using the Wechsler’s Abbreviated Scale of Intelligence (WASI-II) or the WISC-IV/V when scores were available from the past 2 years. Full-scale scores have a mean of 100 and SD of 15

^d^Socioeconomic status (SES) was measured using the MacArthur SES Ladder

^e^Handedness was measured using the augmented 15-item index of the Edinburgh handedness inventory

^f^Musical ability was measured using the global accuracy score on the Montreal Battery for Evaluation of Musical Abilities (MBEMA)^63^

^g^SRS-II: Social Responsiveness Scale. Range: higher scores mean poorer skills

^h^CCC-2: Children’s Communication Checklist. Details provided in Supplementary text

^i^PPVT-4: Peabody Picture Vocabulary Test. Details provided in Supplementary text

^j^Family Quality of Life was measured using the Beach Questionnaire. Details provided in Supplementary text

^k^VABS: Vineland Adaptive Behaviour Scales. Estimated v-scale scores with mean of 15 and SD of 3 for the gross motor skills, fine motor skills and maladaptive behaviours subdomains are reported. Scores between 12 and 18 estimate performance in the average range

In the second session, participants completed a 20-minute MRI scan in a 3 Tesla Siemens Magnetom TimTrio scanner with a 32-channel head coil at the MNI. During this scan, participants were asked to fixate on a cross-hair on the screen. Resting-state BOLD echo-planar images were obtained in 38 slices with a 3.5 mm^3^ voxel resolution, covering the entire brain (TR = 2340 ms, TE = 30 ms, matrix size, 64 × 64; field of view (FOV), 224 mm; flip angle 90°). One hundred and forty volumes were obtained in 5 minutes 32 s. Participants also completed a high-resolution sagittal T1-weighted anatomical scan with a voxel resolution of 1 mm^3^ and an acceleration factor of 2. Participants with their parents underwent a detailed orientation procedure before the MRI scan to ensure comfort and compliance and to maximize good quality outcomes^64^. Audio-visual media aids and mock scanner trials were used in most cases to motivate the participants. Participants’ wakefulness and motion during the actual scans was monitored using an MRI-compatible infra-red camera.

### Randomization and blinding

Fifty-one participants were randomized to MT (*n* = 26) or NM (*n* = 25) using the covariate-adaptive method^65^ where the first 20 participants were randomized using simple coin toss and remaining 31 by the MinimPy software (http://minimpy.sourceforge.net/) by the first author (M.S.), who was not involved in assessing behavioural outcomes. MinimPy is a free, open-source, desktop program implemented in Python, which allows random allocation of subjects to treatment groups in a clinical trial using a stochastic covariate-adaptive minimization algorithm^66^. The success of randomization was assessed by comparing baseline similarity of intervention groups. All other assessors and authors were blind to group allocation information. Our attempt to blind parents (who assessed parent-rated outcomes) was only partially successful, with 31 out of the 51 parents reporting awareness of group allocation. Data were independently double entered to ensure accuracy and stored on an electronic server with restricted, password-controlled access.

### Interventions and fidelity

Both interventions (Fig. S1) involved 45-minute individual weekly sessions conducted over 8–12 weeks by the same accredited therapist (M.T.) using established approaches. Using a child-centric approach, MT made use of musical instruments, songs and rhythmic cues while targeting communication, turn-taking, sensorimotor integration, social appropriateness and musical interaction^47,67–69^. NM was designed as a structurally matched “active comparison” play-based intervention to control for non-specific factors, such as positive treatment expectancies, intervention support, therapist attention and emotional engagement. Both interventions were conducted in the same setting and targeted similar outcomes using theoretically motivated approaches^70^ such as creating a shared experience, building meaningful relationships and emphasizing self-expression^71^ through the use of varied activities targeting common goal such as verbal and social communication, multisensory integration and emotional regulation (SI Table S1). The primary difference was the use of music as a central component in MT. All sessions were video-recorded to assess treatment fidelity^72^ (Supplementary Information).

### Outcomes

#### Behavioural outcomes

Primary behavioural outcomes included a social communication battery consisting of the CCC-2 to measure pragmatic communication, SRS-II to measure symptom severity and PPVT-4 to measure receptive vocabulary. Secondary outcomes were FQoL and the maladaptive behaviours subdomain of the VABS. Outcomes were selected to provide both direct and parent-reported evaluations of treatment-related change using measures that have good psychometric properties, limited practice effects and applicability to a wide range of individuals^73,74^ and were collected at baseline and post-intervention for *n* = 50 participants (Supplementary Information).

#### Statistical analysis

Behavioural outcomes were analysed by fitting linear mixed-effects models (LMEMs) with restriction maximum-likelihood estimation to cope with missing data, inhomogeneity of dependent-variable-variance across factor levels and unequal group size. LMEMs with treatment group (MT, NM), timepoint (baseline, post-intervention) and their interaction as well as participant intercept as random effect were estimated for all primary and secondary behavioural outcomes^75^. Prior to analysis, data were checked for normality. A group×timepoint interaction indicating a change in MT vs. NM post-intervention at *P* < .016 (Bonferroni-corrected from alpha-level of P = .05 to account for three primary behavioural outcomes) was considered significant. Clinical significance was limited to changes from baseline to post-intervention within MT or significant difference between MT and NM post-intervention as confirmed by post hoc Tukey tests at alpha-level of *P* = .05. An intention-to-treat analysis was carried out, whereby missing data from any drop-out participants was replaced with data at baseline. Both unstandardized (beta-coefficients and mean difference) scores^76^ and standardized effect sizes (standardized mean difference, Cohen’s *d*) are reported since standardized effect sizes are often influenced by study design and complexity of models used. Standardized effects sizes are calculated as the difference in change scores between groups divided by the pooled within- and between-group standard deviation^77^. The unstandardized measure is a simple effect size (with 95% confidence intervals (CIs)) in terms of mean difference and does not depend on variance estimates^78^. All statistical analyses were done in R v3.3.4^79^.

#### Neuroimaging outcomes

Primary neuroimaging outcomes were intrinsic functional brain connectivity of fronto-temporal brain networks measured using rsfMRI at baseline and post-intervention. RSFC methods provide an approach for investigating how musical engagement may alter functional connectivity among several brain regions. RSFC metrics of inter-regional correlations specifically afford the advantage of being task-independent, have high test–retest reliability and provide reliable estimates of brain functional connectivity^80^. RSFC metrics also have limited practice effects and may provide an objective method to measure response-to-intervention^40^. Here we tested the extent to which music alters fronto-temporal RSFC in six fronto-temporal seed regions.

#### Image preprocessing

Resting-state images were first preprocessed using FSL (v. 5.0.9; www.fmrib.ox.ac.uk, FMRIB’s Software Library, FMRIB, Oxford, UK)^81,82^ via the SeeBARS pipeline developed at the Center for Research on Brain, Language and Music^83^. Image preprocessing steps consisted of removal of the first five volumes in each scan series as well as removal of non-brain tissue using BET^81^, slice-time correction, motion correction (using a six-parameter affine transformation implemented in FLIRT, global intensity normalization, spatial smoothing (Gaussian kernel of FWHM = 6 mm), temporal high-pass filtering (100 s) and temporal band-pass filtering (0.01–0.1 Hz). To achieve the transformation between the low-resolution functional data and standard space (MNI152: average T1 brain image constructed from 152 normal subjects), two transformations were performed: (1) T2*-weighted image to T1-weighted structural image (using a 7 degree of freedom (DOF) transformation) and (2) T1-weighted structural image to average standard space (using a 12 DOF linear affine transformation, voxel size = 2 × 2 × 2 mm^3^). In addition, physiological noise was removed using the method described by Vahdat and colleagues^83^. The global signal was calculated by averaging the time series over all voxels in the brain. In total, 18 nuisance regressors were used: white matter, cerebrospinal fluid, global signal and their derivatives, and six motion parameters and their derivatives in the first-level analysis^84,85^. Additional motion scrubbing was done using guidelines in Power et al. (2012)^86^. Volumes with framewise displacement (FD) = 0.5 mm or DVARS = 50 (the spatial root mean square of the data after temporal differencing) were masked from whole-brain analysis. Participants with >35% volumes censored at either timepoint were excluded from further analysis (*n* = 6, MT = 2, NM = 4).

#### Statistical analysis

Seeds were defined as 6 mm spheres around coordinates in the left and right Heschl’s gyrus (HG; ±46 −18 10), left and right inferior frontal gyrus (±50 18 7) and left and right temporal pole (TP; ±38 10 −28; Fig. S3). These seeds are known to anchor fronto-temporal networks involved in language and communication and altered in ASD^87^. The timeseries for each of the six seeds was used to generate individual participant-level maps using whole-brain general linear models at baseline and post-intervention. The unthresholded participant-level maps were then entered into a group-level analysis. To assess potential differences between groups at baseline, independent sample *t* tests were computed for maps from all seeds. No baseline differences between groups on any of the six RSFC networks was found (all *P* > .05). To compare groups post-intervention, we used adjusted analysis of covariance (ANCOVA) with post-intervention RSFC as the dependent variable and intervention group, mean-centred baseline RSFC, age, IQ and mean FD^86^ as covariates. Using covariate-adjusted ANCOVA models are more powerful as they can account for baseline imbalance and correlation between baseline and post-intervention measures, increase statistical power and minimize biases^88–92^. *Z*-scores of parameter estimates were used to measure connectivity strength. In RSFC maps where a difference between groups was observed, we evaluated whether post-intervention RSFC was related to improvement in behavioural outcomes (measured by difference scores) in a whole-brain analysis. *Z*-statistics were extracted for each participant from the post-intervention RSFC maps and used in a linear regression model to evaluate strength of the association between RSFC and behavioural improvement. To account for multiple comparisons, random-field theory using a cluster-forming threshold of *P* < .001 was applied^93^. To account for six seeds, a Bonferroni correction was used and a final alpha-level of *P* = .00016 was used for significance testing. All locations are described in MNI coordinates.

## Results

### Participants

One hundred and eleven children meeting diagnosis for ASD were screened in the community, of which 60 did not meet study criteria or declined to participate (further details are provided in Fig. 1). Fifty-one participants in the age range 6–12 years (mean age = 10.25 years, 8 females) were assessed at baseline and randomly assigned to music (MT; *n* = 26) or non-music (NM; *n* = 25) intervention groups (Fig. 1,S1). Assessment at baseline consisted of two sessions: (1) first, detailed demographics, diagnostic reports and baseline measurements of behavioural outcomes were obtained (Table 1). (2) In the second session, anatomical and rsfMRI brain images were obtained on a 3 Tesla MRI scanner. Participants did not differ at baseline on age, sex, language, motor skills, IQ, SES or musical ability and completed an average of 10.3 therapy sessions (*n* = 5 participants had <10 sessions) during the study (Table 1). Fifty participants completed follow-up assessments with one drop-out whose baseline data was used for analysis.

### Treatment fidelity

Treatment fidelity^72^ of delivery of both interventions was assessed using 103 out of the 527 video-recorded intervention sessions by two raters blind to session order, not involved in the trial and demonstrating high inter-rater reliability (intraclass correlation coefficient = 0.91, *P* < .001). There was high adherence to treatment protocols, process fidelity (80–100% with no difference between groups; *P* = .24) and content fidelity (>75% with no difference between groups; *P* = .16) of delivered intervention with no difference in implementation fidelity across MT and NM (Supplementary Information).

### Behavioural outcomes

**Fig. 2:**
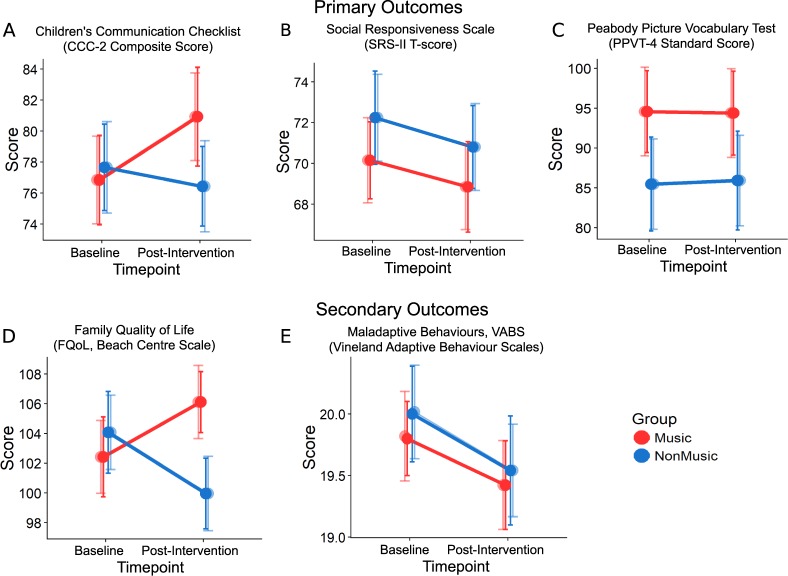
Behavioural outcomes. Line graphs represent effects of Music (MT) vs. Non-music (NM) intervention at baseline and post-intervention timepoints for primary (top panel) and secondary (bottom panel) behavioural outcomes. **a** Higher CCC-2 composite scores for Music Group at Post-intervention (group×timepoint: *β* = −1.35, *P* = .01). **b**, **c** No significant interactions for SRS-II (*β* = −0.04, *P* = .92) and PPVT-4 (*β* = 0.15, *P* = .78). **d** Better FQoL (family quality of life) in the Music Group at post-intervention (group×timepoint: *β* = −1.90, *P* = .01). **e** Reduced VABS Maladaptive Behaviours for both MT and NM post-intervention (*β* = 0.22, *P* = .01). MT is shown in red and NM in blue; darker shades represent observed values and lighter shades represent predicted values. Errors bars represent standard error (SE)

Using LMEMs, we found that MT, relative to NM, showed improvements in communication on the CCC-2, indicated by a significant group×timepoint interaction corrected for multiple outcomes (*β* = −1.35, *P* = .01; MT_Post-intervention_–MT_Baseline_: post-hoc Tukey test, *t* = 1.43, *P* = .024, Fig. 2a). The simple effect size calculated as a mean difference between MT and NM scores from baseline to post-intervention was 4.84 (95% CI: 0.76–8.92) with a larger proportion in the MT group (15/26) compared to the NM group (5/24) showing an improvement. An exploratory analysis of scaled subtests of the CCC-2 (not subjected to correction for multiple comparisons) revealed that these differences stemmed from tests of structural language, Speech (*P* = .01) and Semantics (*P* = .046); a pragmatics subtest, Inappropriate Initiations (*P* = .006); and two autism-relevant subtests (not included in the CCC-2 composite): Social Relations (*P* = .048) and Interests (*P* = .02). There was no group×timepoint interaction on the SRS-II^55^ *t*-scores (*β* = −0.04, *P* = .92; mean difference = 0.65, 95% CI:−3.25 to 4.1), or PPVT-4^62^ standard scores (*β* = 0.15, *P* = .78; mean difference = 0.03, 95% CI:−4.32 to 4.38; Fig. 2b, c). There was, however, a significant group×timepoint interaction on parent-reported FQoL (*β* = −1.9, *P* = .01, Fig. 2d) with mean difference = 7.06 favouring MT (95% CI: 0.79 to 13.33) even though no post hoc tests were significant. Additionally, both groups showed reduction in maladaptive behaviours on the VABS post-intervention (*β* = 0.22, *P* = .01, Fig. 2e, Table 2, SI Table S2).

**Table 2:** Behavioural outcomes

Outcomes	Observed values						Effect size		
	Music			Non-music			Mean difference	±95% CI	Standardized effect size (*d*)
	*n*	Mean	±95% CI	*n*	Mean	±95% CI			
*Primary outcomes*									
CCC-2							4.84	4.08	0.34
Baseline	25	76.84	5.64	23	77.65	5.45			
Post-intervention	24	80.46	6.43	23	76.43	5.02			
Changes from baseline	25	3.62	0.78	23	−1.22	−0.43			
SRS-II							0.65	3.45	0.06
Baseline	26	70.15	3.68	25	72.24	4.47			
Post-intervention	26	69.36	4.39	25	70.8	3.98			
Changes from baseline	26	−0.79	0.71	25	−1.44	−0.49			
PPVT-4							0.03	4.35	0.00
Baseline	26	94.57	10.05	25	85.48	11.52			
Post-intervention	26	95.04	10.66	25	85.92	12.11			
Changes from baseline	26	0.47	0.61	25	0.44	0.59			
*Secondary outcomes*									
FQoL							7.06	6.27	0.57
Baseline	26	102.42	5.25	25	104.08	5.39			
Post-intervention	26	105.36	3.86	25	99.96	4.65			
Changes from baseline	26	2.94	−1.39	25	−4.12	−0.74			
VABS-MB^a^							0.08	0.65	0.04
Baseline	26	19.8	0.59	24	20	0.74			
Post-intervention	26	19.42	0.71	24	19.54	0.86			
Changes from baseline	26	−0.38	0.12	24	−0.46	0.12			

*CCC-2* Children’s Communication Checklist Composite score, *SRS-II* Social Responsiveness Scale T-Score, *PPVT-4* Peabody Picture Vocabulary Test Standard score, *FQoL* Family Quality of Life total score measured using the Beach Centre Scale, *CI* confidence interval

^a^VABS-MB Vineland Adaptive Behaviour Scales–Maladaptive behaviour subdomain v-scale score. Scores <18 are average, scores of 18–20 are elevated and scores 21–24 are clinically significant

### Brain connectivity outcomes

**Fig. 3:**
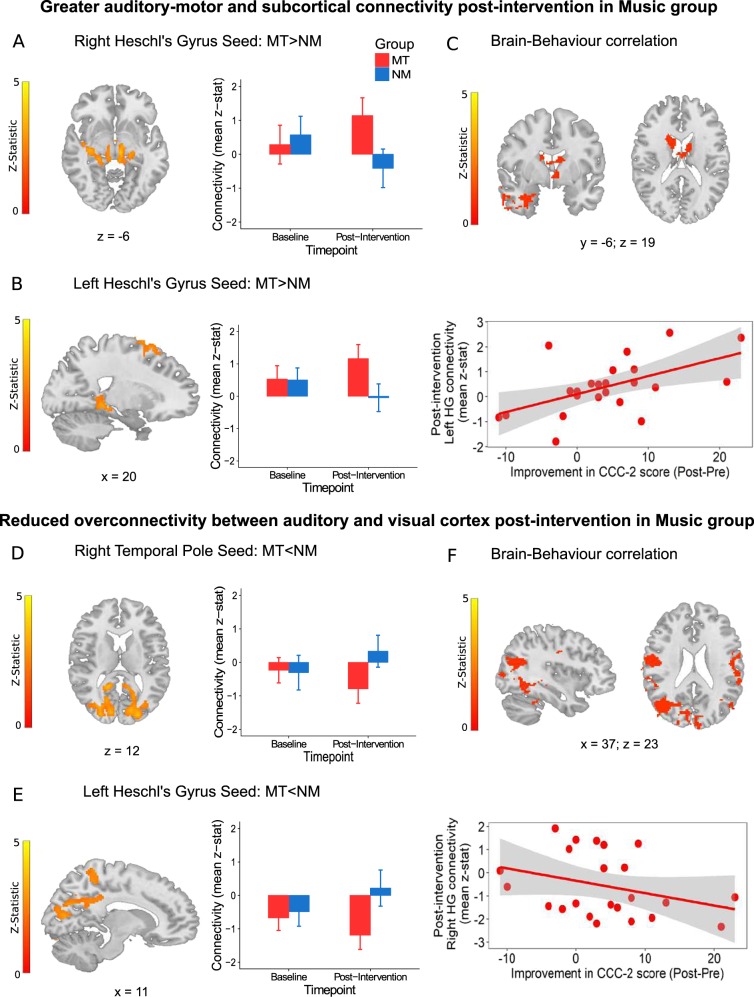
Brain functional connectivity outcomes and correlation with behavioural improvement. The top panel shows regions of increased resting-state functional connectivity (RSFC) post-intervention in the Music (MT) vs. Non-music (NM) groups between **a** Right Heschl’s gyrus seed and subcortical regions such as the hippocampus and thalamus (*z* = 3.94, *P* < .0001) and **b** left Heschl’s gyrus seed and fronto-motor regions (*z* = 3.16, *P* < .0001). **c** Connectivity between auditory and subcortical thalamic and striatal regions post-intervention is directly related to improvements in communication measured using the change in CCC-2 composite score in MT (*z* = 3.57, *P* < .0001). The bottom panel shows regions of decreased RSFC post-intervention in MT vs. NM groups between **d** right temporal pole seed and occipital regions (*z* = 4.01, *P* < .00001) and **e** left Heschl’s gyrus seed and bilateral calcarine and cuneus regions (*z* = 3.39, *P* < .00001). **f** Connectivity between auditory and visual sensory cortices post-intervention is inversely related to improvements in communication measured using the change in CCC-2 composite score in MT (*z* = 3.64, *P* < .001). MT is shown in red and NM in blue. Errors bars represent standard error (SE). All brain images are presented in radiological convention and coordinates are in MNI space

There were no baseline differences between groups on any of the six RSFC networks (all *P* > .05). Using covariate-adjusted ANCOVA models, we found greater RSFC post-intervention in the MT group compared with NM between auditory seeds (left and right HG) and striatal and motor regions (right HG: *z* = 3.94, *P* = .000019, left HG: *z* = 3.79, *P* = .00009, Fig. 3a, b, SI Table S3) and reduced RSFC in MT between auditory seeds (left HG and right TP) and visual regions (left HG: *z* = 3.39, *P* < .00001, right TP: *z* = 4.01, *P* < .00001, Fig. 3d, e, SI Table S3).

To evaluate whether changes in RSFC were related to improvements in behavioural outcome, we tested whole-brain models with CCC-2 improvement (CCC-2_Post-Intervention_−CCC-2_Baseline_) as covariate of interest for the three seeds (left HG, right HG and right TP) where significant differences between groups was found. Greater RSFC post-intervention between left HG and subcortical thalamic and striatal regions was related to greater improvement on CCC-2 scores (*z* = 3.57, *P* < .0001, Fig. 3c). Lower post-intervention RSFC between right HG and visual areas was related to greater improvement in CCC-2 scores (*z* = 3.64, *P* < .001, Fig. 3f).

## Discussion

Individuals with ASD have a unique profile of strengths amid limitations, which can be harnessed to design treatment paradigms that improve functional outcomes^94^. Given their universal appeal, intrinsic reward value and ability to modify brain and behaviour, musical activities have been proposed as a potential strength-based rehabilitation tool for ASD^22,36,95^. In the current trial, we demonstrate that 8–12 weeks of music intervention can indeed alter intrinsic brain connectivity and improve parent-reported outcomes in social communication and FQoL in school-age children with ASD.

Improvements in social communication were found on the CCC-2 from baseline to post-intervention in MT vs. NM, with a medium-sized positive effect (*d* = 0.34). Improvements were specific to pragmatics, reduction of inappropriate initiations and better social relations and interests. These findings are consistent with the idea that music employs a structured approach to social communication, which may otherwise be hindered by sensory and social difficulties^12,36^. Despite being modestly sized, these effects are highly specific to MT given the comparable structure of the control intervention and may have promising clinical and policy implications^96^. No MT-specific improvements were found on SRS-II or PPVT-4. Despite convergence between the SRS-II and CCC-2 and similar susceptibility to assessor-blinding biases, the SRS-II is a measure of ASD symptom severity, whereas the CCC-2 is a pragmatic communication measure, indicating limited effects of MT on reducing ASD symptom severity or improving receptive vocabulary.

We also found a positive effect of MT on FQoL (*d* = 0.57). The family is the primary support system for individuals with ASD throughout their lifespan. However, parents of children with ASD experience high levels of stress that can negatively impact well-being^97^, making FQoL a critical component in evaluating treatment outcomes. Although both groups received a form of interventional support, only parents of children in MT reported increases in FQoL, particularly on items on family interaction, cohesion and coping and benefits of disability-related supports.

Recently, Bieleninik and colleagues^98^ published a multicentre trial of improvisational music therapy for children aged 4–7 years and found no reductions in ASD symptoms on the ADOS Social Affect domain after 5 months of therapy, compared to standard care. The authors suggest that this could be due to variability across therapists, clinical assessors^99^ and the choice of ADOS as outcome measure, particularly because no previous well-controlled and blinded intervention studies^3^ have found treatment effects on the ADOS. While this trial demonstrates the global feasibility of implementing music therapy in large-scale international settings, it also indicates the importance of choosing appropriate outcome measures for psychosocial interventions in heterogeneous neurodevelopmental populations. The focus should not only be on symptom reduction but also on overall quality of life and functional improvements. In turn, outcomes that are malleable through intervention can inform future targets of research.

To complement behavioural improvements, we present the first evidence that music intervention alters functional brain activity in ASD leading to functional communication gains. Specifically, MT, relative to NM, increased functional connectivity between bilateral primary auditory cortex and subcortical and motor regions (often reduced in ASD)^100^ and reduced over-connectivity between auditory and visual-association areas^38^. Importantly, changes in brain connectivity were related to improvements in children’s communication skills after MT (Fig. 3c–f).

Brain connectivity in ASD has often been conceptualized as a trade-off between bottom–up and top–down processing. However, it is still not clear whether an increased reliance on bottom–up sensory processing and hence, sensory over-connectivity, is a cause or consequence of atypical top–down cortical modulation^41,46^. As a result, social communication impairments may result from alterations not just in the brain’s “social” network” but in domain-general disconnections in sensorimotor and cognitive functions, which are building blocks of later social skills^46^. In line with this idea, we find that engaging in musical activities can directly influence auditory–motor connections in the brains of children with ASD similar to effects of musical training in neurotypical populations^27,30,31^. Previous studies have reported that early motor difficulties are often predictive of later social communication impairments in ASD^101^. Thus interventions targeting motor skills may impact later social outcomes. It is important to note that our participants did not exhibit significant motor deficits and that the two intervention groups did not differ in their range of motor skills (Table 1). Thus any gains observed in auditory–motor connectivity in the MT group are not driven by group differences in motor skills and are specific to the music intervention. Furthermore, our findings show that music might play a modulating role in reducing the over-connectivity between sensory cortices, subsequently improving communication processes^102,103^. In light of mechanisms of music-induced neuroplasticity introduced earlier, our findings support bottom–up integration of sensorimotor brain networks leading to improved social functioning rather than top–down music-based reward^36^. Music interventions may thus have a positive influence on social functioning, possibly though modulation of domain-general sensory and cognitive processes, which are often atypical ASD^41,46^. Future research should focus on better understanding the neural mechanisms underlying music-related changes in brain connectivity and its impact on social behaviour.

Evidence-based behavioural and psychosocial interventions for school-age children have received limited attention^3^. Neuroscience-informed support for such interventions offers the opportunity to integrate brain development with behavioural approaches, allowing development of individualized treatment paradigms^104^. A strength of the current study is the use of neuroimaging to support improvements in behavioural outcomes resulting from MT. Consequently, the sample size (*n* = 51) is quite modest. Future work should focus on identifying individuals whose profiles may benefit most from music and integrate neuroimaging in multisite trials of such interventions. Inclusion of more direct observation-based outcomes and the role of mediators and moderators (e.g. quality of therapeutic relationship, cognitive, language and motor profiles, symptom level and musical interest of the participant) on short- and long-term outcomes will also be crucial to further the evidence base for music-based interventions.

In conclusion, the present study demonstrates that 8–12 weeks of music intervention (relative to non-music behavioural intervention) can improve parent-reported social communication, FQoL and intrinsic brain connectivity in school-age children, thus supporting the use of music as a therapeutic tool for individuals with ASD.

## Electronic supplementary material

Supplementary Information
