# Supplementary material for: Music improves social communication and auditory–motor connectivity in children with autism
Source: Transl Psychiatry. 2018 Oct 23;8:231. doi: 10.1038/s41398-018-0287-3 (PMC6199253; doi:10.1038/s41398-018-0287-3)

Supplementary Information (SI) Appendix

1. Intervention design

Intervention groups consisted of Music or non-music therapy. Both groups were 45-minute one-on-one sessions conducted weekly for 8-12 weeks by a certified music therapist using established approaches. The primary difference in the two interventions was the use of music as a reinforcer and central component in MT and its conspicuous absence in NM. But common overarching themes in both intervention settings included shared experience, focus on building meaningful relationships and emphasis on self-expression through a bottom-up integration of sensorimotor skills and non-verbal communication. NM was particularly designed as structurally matched "active comparison" group to control for non-specific factors such as positive treatment expectancies, intervention support, therapist attention, emotional and mental engagement and in particular to allow for reliable interpretations of the role of music as the "active ingredient" that would underlie any treatment effects. 9-11 activities were used in each intervention targeting similar goals (Table S1). Consistent with the recommendations of both music and behavioural therapy, the goal of the above interventions was to integrate sensorimotor approaches with expression through verbal or nonverbal communication and relationship-building approaches. The therapist (MT) and the first author (MS) developed detailed and extensive guidelines to ensure adherence to each of the interventions. Each session had a defined structure with a 'hello' greeting, followed by selection of activities using a visual schedule. There were 4 activities per session. A session log was used to ensure that each child participated in each activity an equal number of times. The sessions ended with a 'goodbye' theme and 'clean up' (Figure S1). All sessions (music and control sessions) were video recorded for post-hoc observational coding by a blind rater.

2. Assessment of fidelity of intervention

Evaluation of intervention fidelity is critical not only to the study of the effectiveness of any intervention but also for its translation into non-experimental settings. In the current trial, intervention fidelity was measured for both music and non-music intervention using study-specific, literature-guided measures in four domains. A fidelity assessment tool was developed in-house prior to the study. After completion of the study, 2 blind raters were trained on a manual for

assessment of treatment fidelity developed to assess the fidelity of the two interventions implement using 103 intervention videos (54 music, 49 non-music).

Inter-rater reliability (IRR)

Treatment fidelity was established using both provider and assessor-based fidelity. The therapist provided implementation fidelity using a checklist after the completion of each session. Two assessors, blind to the session identity and not involved in the actual trial, coded video recordings of 103 sessions (2 from each participant) using the same in-house fidelity checklist to assess process and content fidelity. IRR was established using 30% of videos that were evaluated by both raters (an average rating for these videos was used in the assessment of overall fidelity) and evaluated using IntraClass Coefficients (ICC). The IRR was high between the two raters (ICC=0.91, p<.001).

During the trial, the therapist reviewed the fidelity checklist before each session and filled it out after each session completed. The therapist used a pre-specified session plan for each participant which contained notes from the previous session and indicated the activity structure for the current session. Sessions were video recorded for subsequent review by the interventionist as well as for assessment of adherence to treatment protocol and assessment of content fidelity using the same fidelity checklist by blind assessors. Because of the dynamic and often individualized nature of dyadic interactions between interventionist and client, systematic assessment of treatment fidelity is often challenging for psycho-social and cognitive-behavioural interventions [1,2]. Our goal was to follow a semi-structured session plan with room for individual adaptation by clearly defining elements that were standardized and those that could be individualized for each participant while monitoring both process and content aspects of each intervention. All sessions were semi-structured and consisted of four pre-selected activities (randomized and counterbalanced across participant and intervention). However, the order of each activity was chosen by the participant. The interventionist was evaluated on whether she appropriately used judgment and discretion, which applied to both music and non-music interventions. Additionally, the fidelity checklist tool assessed the implementation of the critical ingredients of evidence-based music therapy as well as behavioural intervention: quality of dyadic interaction, use of reinforcement (verbal or musical), relationship-building, and focus on communication.

1)Adherence: Program adherence was defined as the number of sessions completed by each participant in both groups as well as the total number and types of activities covered, which were recorded using weekly reports by the interventionist. A total of 527 (MT=273, NM=254) intervention sessions were delivered between April and December 2016. Participants completed an average of 10 (out of 12 total) intervention sessions in each group with only n=5 participants completing fewer than 10 sessions. In the MT group, participants completed each of the 11 activities three or four times over the course of therapy. In the NM group, participants completed each of the 9 activities 4-5 times.

2) Process fidelity (PF): PF was defined as delivery of key theoretical concepts of the structured intervention using yes/no categories using a 5-item checklist. PF ranged between 4-5 for all sessions evaluated (80-100% process fidelity), indicating high process fidelity and did not differ across interventions (p=.24).

3) Content Fidelity (CF): CF was defined as the establishment of a therapeutic relationship between the participant and the interventionist. CF was measured using quality of delivery and participant responsiveness as well as the theoretical principles underpinning the two interventions using a 6-item checklist for each activity. Each item was scored on a likert-scale of 0 (Never/Rarely) to 3 (Almost always). An average of 60% CF was achieved overall across all six items and four activities in both interventions. The lowest scoring modules were prompting and shaping behaviour. Given the wide age range and range of ability of the participants, this was a dynamic measure often adapted to the needs of each individual child and the scoring of fidelity likely suffered for it. The other four items (verbal praise, initiation, facilitation, playing together), were rated highly for content fidelity (>75%). The average content fidelity did not differ between the intervention groups (p=.16)

4) Program differentiation was evaluated using a single item to differentiate between the music and non-music intervention: “Music was central to this activity” scored as yes or no. While the music intervention relied on music-based communication at its core, the non-music intervention made use of social interaction and non-music aids to achieve similar goals.

Overall, the interventionist successfully delivered structured intervention content and there was no difference in implementation fidelity across the two interventions across the various fidelity domains.

SI. 2. Behavioural outcome measures

Primary behavioural outcomes

Children's Communication Checklist (CCC-2)

The CCC-2 is a parent/caregiver-administered 70-item rating scale used to measure children's social communication skills in 10 domains [3]. This test focuses on assessment of non-verbal communication, pragmatics, as well as aspects of language structure and discourse which are often impaired in ASD [4]. The standard general communication composite standard score was used in the present study as a measure of the child's general pragmatics and communication ability. Scores on the general composite of the CCC-2 have a mean of 100 (SD=15) and have very good test-retest reliability (r=0.93) and have been used in previous studies of social-communication as an outcome measure [5]. Higher scores indicate better social-communication skills. Scores were not obtained from n=3 participants who did not have sentence-level speech.

Social Responsiveness Scale-II (SRS-II)

The SRS-II [6] is a 65-item parent-rated measure encompassing dimensions of interpersonal behaviour, communication and repetitive behaviour in five domains. The SRS-II is often used as a measure of symptom severity in autism treatment studies. It has good psychometric properties (test-retest reliability >.88). The SRS T-scores have a mean of 500 (SD=10). Higher scores indicate poorer social communication skills.

Peabody Picture Vocabulary Test (PPVT-4)

The PPVT-4 is a short, standardized measure of one-word receptive vocabulary [7]. The test requires the participant to choose one of four color pictures on a page that is named by the examiner. As such, the PPVT-4 does not necessitate spoken language and has norms for 2–90 year olds, allowing use of the same test across individuals of different ages and varying cognitive and language abilities. Additionally, the PPVT-4 comes with 2 separate forms, minimizing practice effects and

making it a useful tool to measure change in outcomes over time. The PPVT-4 has a mean of 100 (SD=15). Higher scores indicate better receptive vocabulary.

Secondary behavioural outcomes

Beach Center Family Quality of Life Scale (FQoL)

The FQoL [8],assesses families' perceptions of their satisfaction with different aspects of family quality of life and consists of a 25-item questionnaire containing five subscales: Family Interaction (the relationships among and between family members), Parenting (the kinds of activities families engage in to facilitate their child's development), Emotional Well-Being (perceptions of stress and support-availability), Physical/Material Well-Being (basic physical needs such as medical support and transportation), and Disability-Related Support (supports across the community contexts of school, work, and home). It has well-validated psychometric properties and can be used in pre-post studies to evaluate family outcomes.

Maladaptive Behaviours (Vineland Adaptive Behaviour scale (VABS)

The maladaptive behaviours subdomain of the VABS [9,10] is used to identify the presence of behavior problems such as challenging internalizing and externalizing behaviours in children up to age 18. The scale is administered as a semi-structured interview to an informant who knows the child well. VABS v-scale scores have a mean of 15, (SD=3). Three categories are used to convey the degree of maladaptive behaviour in an individual corresponding to v-scale scores- Below 18: Average, 18-20: Elevated, 21-24: Clinically significant.

Supplementary References

1 Thomeer ML, Rodgers JD, Lopata C, Nelson AT, Booth AJ. Achieving and maintaining intervention fidelity using a three-component implementation model in a comprehensive school-based psychosocial treatment for children with high-functioning ASD. In: *Autism Spectrum Disorders: Early Signs, Intervention Options and Family Impact*. Nova Science Publishers, Inc., 2015, pp 129–155.

2 Geretsegger M, Holck U, Bieleninik Ł, Gold C. Feasibility of a trial on improvisational music therapy for children with autism spectrum disorder. *J Music Ther* 2016. doi:10.1093/jmt/thv038.

3 Bishop DVM. Development of the Children's Communication Checklist (CCC): A Method for Assessing Qualitative Aspects of Communicative Impairment in Children. *J Child Psychol Psychiatry* 1998; **39**: 879–891.

4 Boucher J. Research review: structural language in autistic spectrum disorder - characteristics and causes. *J Child Psychol Psychiatry* 2012; **53**: 219–33.

5 Adams C, Lockton E, Freed J, Gaile J, Earl G, McBean K *et al.* The Social Communication Intervention Project: a randomized controlled trial of the effectiveness of speech and language therapy for school-age children who have pragmatic and social communication problems with or without autism spectrum disorder. *Int J Lang Commun Disord* 2012; **47**: 233–44.

6 Constantino JN, Davis SA, Todd RD, Schindler MK, Gross MM, Brophy SL *et al.* Validation of a Brief Quantitative Measure of Autistic Traits: Comparison of the Social Responsiveness Scale with the Autism Diagnostic Interview-Revised. *J Autism Dev Disord* 2003; **33**: 427–433.

7 Dunn LM. Peabody picture vocabulary test-revised (PPVT-R). Forms L and M. 1981.

8 Park J, Hoffman L, Marquis J, Turnbull AP, Poston D, Mannan H *et al.* Toward assessing family outcomes of service delivery: validation of a family quality of life survey. *J Intellect Disabil Res* 2003; **47**: 367–84.

9 Mouga S, Almeida J, Café C, Duque F, Oliveira G. Adaptive Profiles in Autism and Other Neurodevelopmental Disorders. *J Autism Dev Disord* 2014. doi:10.1007/s10803-014-2256-x.

10 Sparrow S, Balla D, Cicchetti D. *Vineland Adaptive Behaviour Scales*. Interview. American Guidance Service: Circle Pines, MN, 1984.

Supplementary Tables

Table S1. Intervention activities

Goal	Music activity	Non-music activity
Verbal Communication	Harmonica, Singing, Pete-the-Cat Story board with song, Recorder,	Pete-the-Cat Story board without song, Finger Puppets, Story book, Play Doh
Reciprocal Social communication	Piano, Xylophone, Pete-the-Cat Story board with song, Djembe	Pete-the-Cat Story board without song, Finger Puppets, Egg Shakers, Darts, Textured Bean Bags
Fine motor skills	Piano, Xylophone, Egg Shakers, Drums	Darts, Play Doh, Lego, Finger puppets,
Multisensory integration	Drums, Handheld Percusion, Xylophone, Melodica	Bubbles, Play Doh, Jigsaw, Lego, Finger Puppets
Emotional regulation	Drums, Singing,	Finger Puppets, Play Doh, verbal interaction during activities

Table S2. Behavioural outcomes: statistical results of linear mixed-effects models

Outcome	Effect	Unstandardized Beta coefficients	- 95% CI	+95% CI	Sigma	P values
Primary outcomes						
CCC-2[a]						
Composite	Intercept	77.98	74.11	81.86	5.08	

	Time	-0.74	-1.76	0.29	5.08	0.16
	Group	0.94	-2.93	4.82	5.08	0.63
	Time X Group	-1.35	-2.37	-0.32	5.08	0.01
SRS-II[b] T-score	Intercept	70.52	67.72	73.32	4.40	
	Time	0.68	-0.19	1.53	4.40	0.12
	Group	-1.00	-3.80	1.80	4.40	0.48
	Time X Group	-0.04	-0.91	0.81	4.40	0.92
PPVT-4[c]						
Standard Score	Intercept	90.09	82.38	97.81	5.55	
	Time	-0.07	-1.15	1.02	5.55	0.90
	Group	4.39	-3.32	12.11	5.55	0.26
	Time X Group	0.15	-0.93	1.24	5.55	0.78
Secondary outcomes						
FQoL[d]	Intercept	103.09	100.04	106.15	8.04	
	Time	0.16	-1.40	1.74	8.04	0.84
	Group	1.07	-1.98	4.13	8.04	0.49
						0.01
	Time X Group	-1.90	-3.46	-0.32	8.04	
VABS[e]						
Maladaptive						
Behaviours	Intercept	19.70	19.21	20.19	0.80	
	Time	0.22	0.06	0.38	0.80	0.01
	Group	-0.08	-0.56	0.41	0.80	0.75
	Time X Group	-0.02	-0.18	0.14	0.80	0.81

[a]CCC-2: Children's Communication Checklist

[b]SRS-II: Social Responsiveness Scale.

[c]PPVT-4: Peabody Picture Vocabulary Test

[d]FQoL: Family Quality of Life measured using the Beach Centre Scale

[e]VABS: Vineland Adaptive Behaviour Scales

Table S3. Brain connectivity outcomes: statistical results by cluster and contrast

Seed	Contrast	Brain Region	Z-statistic	Coordinates			P values
				x	y	z	
Right Heschl’s Gyrus (HG)	Music (n=24)> Non-music (n=21)	Left Thalamus/Brainstem	3.94	-8	-22	-6	1.940E-05
		Left Brainstem	3.44	-8	-42	-14	1.940E-05
		Right Hippocampus	3.44	32	-22	-8	1.940E-05
		Left Hippocampus	3.44	-32	-22	-8	1.940E-05
		Right Thalamus	3.51	8	-36	2	1.940E-05
Left HG	Music (n=24)> Non-music (n=21)	Right Parahippocampal Gyrus	3.42	18	-36	-6	5.960E-08
		Left Lingual Gyrus	3.12	-24	-60	-10	5.960E-08
		Left Hippocampus	3.09	-20	-40	2	5.960E-08
		Left Supplementary Motor Area	3.79	-6	10	72	9.040E-05
		Left Supplementary Motor Area	3.54	-2	16	66	9.040E-05
		Right Superior Frontal/Supplementary Motor area	3.16	20	18	64	9.040E-05
		Right Superior Frontal/Supplementary Motor area	3.06	18	8	72	9.040E-05
		Right Superior Frontal/Supplementary Motor area	3.05	4	4	70	9.040E-05
Right Temporal Pole (TP)	Music (n=24)< Non-music (n=21)	Left Lingual Gyrus	4.01	-16	-76	0	2.210E-06
		Left Inferior Temporal Gyrus	3.71	-44	-74	-4	2.210E-06
		Left Middle Occipital Gyrus	3.69	-26	-90	4	2.210E-06

		Left Calcarine Sulcus	3.61	-16	-78	8	2.210E-06
		Right Lingual Gyrus	3.79	20	-84	-6	2.280E-05
		Right Lingual Gyrus	3.63	22	-82	-14	2.280E-05
		Right Cuneus	3.73	12	-86	8	2.280E-05
Left HG	Music (n=24)< Non-music (n=21)	Right Calcarine Sulcus	3.39	10	-78	10	2.460E-10
		Right Cuneus	3.32	10	-70	26	2.460E-10
		Right Cuneus	3.3	6	-74	26	2.460E-10
		Right Postcentral Gyrus	3.27	40	-32	48	2.460E-10
		Left Precuneus	3.06	-2	-74	36	2.460E-10
		Left Cuneus	3.02	0	-80	36	2.460E-10
		Right Inferior Occipital Gyrus	3.17	40	-82	-16	2.490E-05
		Right Inferior Occipital Gyrus	3.1	38	-76	-12	2.490E-05
		Left Superior Parietal Lobule	3.6	-18	-48	68	3.260E-04

Supplementary Figures

Fig. S1. Intervention design: Both interventions, Music and Non-Music consisted of 45-minutes sessions. Each session had a defined structure with a 'hello' greeting, followed by selection of 4 activities per session using a visual schedule. A session log was used to ensure that each child participated in each of 9-11 activities an equal number of times. The sessions ended with a 'goodbye' theme and 'clean up'. Both interventions targeted similar skills with and without the use of music.

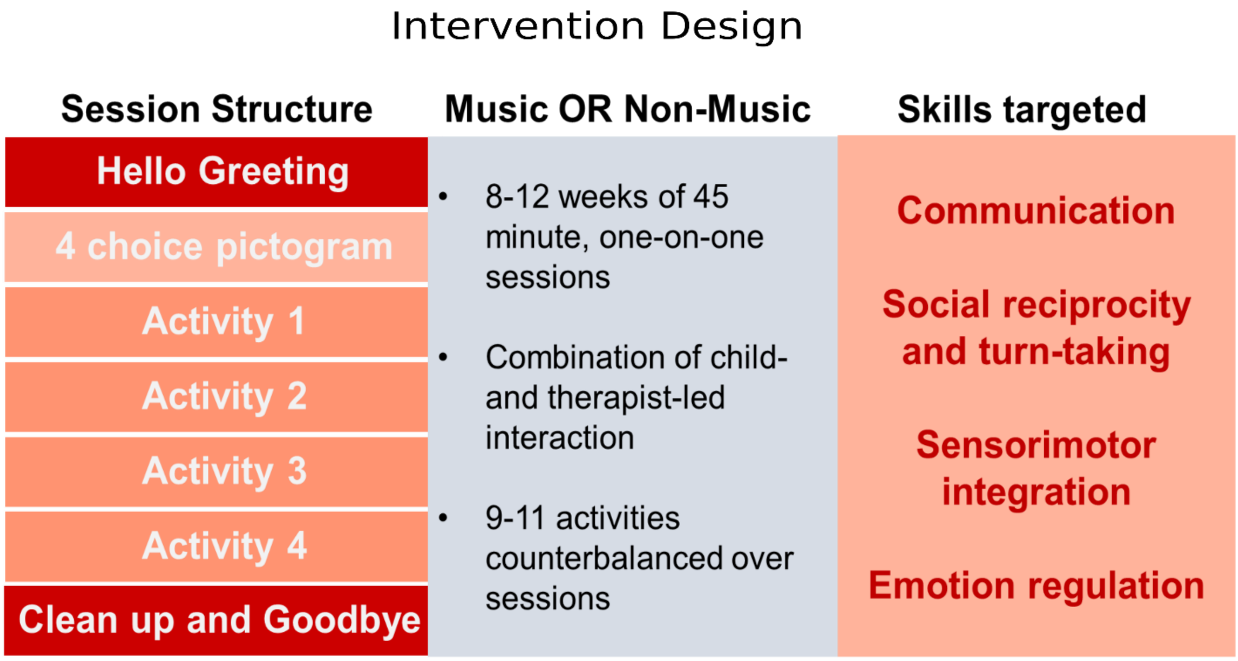

Fig. S2. Seed regions: 6 seeds were used for resting-state functional connectivity analyses. These seeds were located in left and right Heschl's gyrus (+/-46 -18 10), left and right inferior frontal gyrus (+/-50 18 7) and left and right temporal pole (+/-38 10 -28). Left hemisphere seeds are indicated in blue and right hemisphere seeds in red. All locations are reported in MNI coordinates.

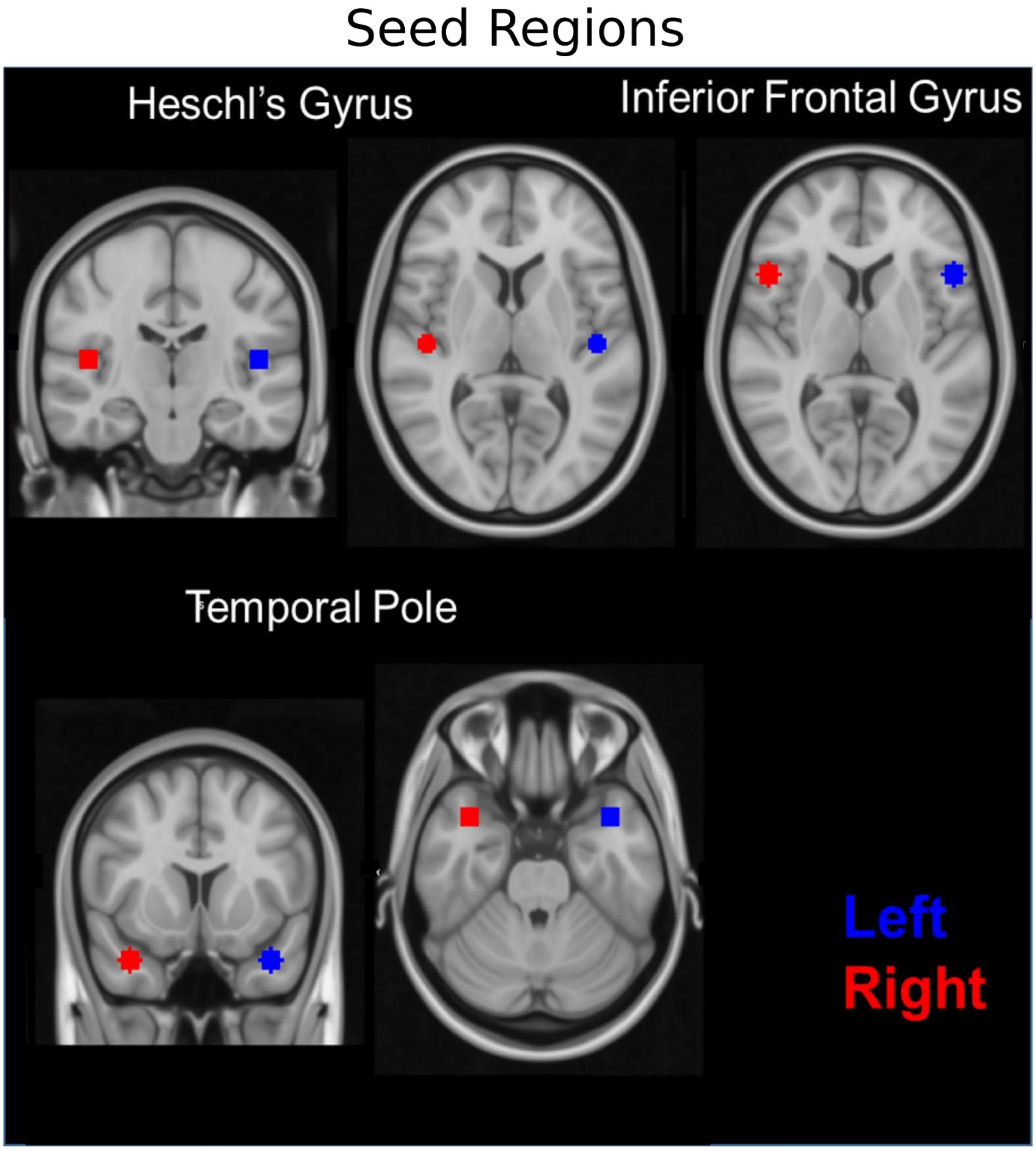

Supplement: Supplementary file 1 — Supplementary Information [file 41398_2018_287_MOESM1_ESM.pdf]
